# Cost-effectiveness analysis of vaccination against COVID-19 in China

**DOI:** 10.3389/fpubh.2023.1037556

**Published:** 2023-03-07

**Authors:** Huixuan Zhou, Ningxin Ding, Xueyan Han, Hanyue Zhang, Zeting Liu, Xiao Jia, Jingjing Yu, Wei Zhang

**Affiliations:** ^1^Department of Physical Fitness and Health, School of Sport Science, Beijing Sport University, Beijing, China; ^2^Key Laboratory of Exercise and Physical Fitness, Ministry of Education, Beijing Sport University, Beijing, China; ^3^School of Government, Wellington School of Business and Government, Victoria University of Wellington, Wellington, New Zealand; ^4^School of Health Policy and Management, Chinese Academy of Medical Sciences and Peking Union Medical College, Beijing, China; ^5^School of Physical Education, North East Normal University, Jilin City, China; ^6^Department of Mathematic Science, School of Sport Engineering, Beijing Sport University, Beijing, China; ^7^Department of Chemical Drug Control, China National Institute for Food and Drug Control, Beijing, China

**Keywords:** COVID-19, vaccination, cost-effectiveness analysis, cost-utility analysis, SARS-CoV-2

## Abstract

**Introduction:**

Since September 2020, Chinese populations aged > 3 years have been encouraged to receive a two-dose inoculation with vaccines against coronavirus disease 2019 (COVID-19). This study aims to evaluate the cost-effectiveness of the current vaccination strategy amongst the general population in mainland China from a societal perspective.

**Methods:**

In this study, we construct a decision tree with Markov models to compare the economic and health consequences of the current vaccination strategy versus a no-vaccination scenario, over a time horizon of one year and an annual discount rate of 5%. Transition probabilities, health utilities, healthcare costs, and productivity losses are estimated from literature. Outcome measures include infection rates, death rates, quality-adjusted life years (QALYs), and costs. The incremental cost-effectiveness ratio (ICER) is then calculated to evaluate the cost-effectiveness of the current vaccination strategy, and both one-way deterministic sensitivity analysis and probabilistic sensitivity analysis (PSA) are applied to assess the impact of uncertainties on results.

**Results:**

Our simulation indicates that compared with a no-vaccination scenario, vaccination amongst the general population in mainland China would reduce the infection rate from 100% to 45.3% and decrease the death rate from 6.8% to 3.1%. Consequently, the strategy will lead to a saving of 37,664.77 CNY (US$5,256.70) and a gain of 0.50 QALYs per person per year on average (lifetime QALY and productivity loss due to immature death are included). The cost-saving for each QALY gain is 74,895.69 CNY (US$10,452.85). Result of the PSA indicates that vaccination is the dominating strategy with a probability of 97.9%, and the strategy is cost-effective with a probability of 98.5% when the willingness-to-pay (WTP) is 72,000 CNY (US$10,048.71) per QALY.

**Conclusion:**

Compared with a no-vaccination scenario, vaccination among the general population in mainland China is the dominating strategy from a societal perspective. The conclusion is considered robust in the sensitivity analyses.

## 1. Introduction

Since late 2019, the novel coronavirus disease 2019 (COVID-19) has spread across 222 countries and regions, causing over 102 million cases and 2.2 million deaths worldwide (1). Its pathogen, SARS-CoV-2, which is mainly transmitted *via* respiratory droplets and contacts, is even more transmissible than the SARS-CoV in 2003 (2).

The pandemic has been effectively controlled under the regulations introduced to restrict public activities in many countries. However, such restrictions also have a side effect on the economy. For example, in China, statistics have shown a productivity loss of 2,646.7 billion CNY (US$383.0 billion) due to the public restrictions in 2020 (3), but the healthcare expenditures for COVID-19-related treatments were only 4.3 billion CNY (US$ 0.6 billion) in 2020 (3). Given these, it would be critical to introduce an intervention that may curb rapid disease transmission without yielding a huge loss to the economy. Therefore, vaccination is probably a potential solution (4, 5).

COVID-19 vaccines were developed quite fast, and many of them have been approved by different countries worldwide. By September 2021, five COVID-19 vaccines developed by Chinese pharmaceutical companies had been authorized in mainland China (6). Three of them are inactivated vaccines, and the other two are a protein subunit vaccine and an adenovirus vaccine. Given the safety and efficacy of the vaccines (7–10), Chinese populations aged > 3 years have been encouraged to take a two-dose inoculation of inactivated vaccines since September 2020. This strategy was made based on the foundation that Chinese pharmaceutical companies have produced 1.4 billion doses of vaccines since 2021, and 570 million of them have been exported overseas (11). Therefore, the supply of vaccines is adequate for a collective vaccination, and the Chinese government does not have to determine the prioritization of vaccination by health risks like many Western countries have been doing.

However, there is still a lack of evidence regarding the cost-effectiveness of the current vaccination strategy from a societal perspective in mainland China. Therefore, this study conducts the cost-utility analysis (CUA) of vaccination against COVID-19 among the general population in mainland China, exploring the influences of vaccination on society. The findings are expected to lay a sound foundation for the decision-making of disease control strategies for the government.

## 2. Methods

### 2.1. Model overview

A decision tree with Markov models is constructed to compare the health and economic consequences of vaccination against COVID-19 vs. a no-vaccination scenario from a societal perspective using the TreeAge Pro Healthcare software (TreeAge Software, LLC.).

In the model, we assume that the targeted individuals are the general population aged over 3 years in mainland China. We believe that this assumption is appropriate because Chinese citizens aged over 3 years have been recommended to become vaccinated by the National Health Commission of China. The decision was mainly made based on the evidence that severe and immediate allergic reactions are the only absolute contraindications of vaccination (6). Moreover, there is a lack of evidence to show the differences in vaccine efficacy and clinical characteristics among various subgroups of the Chinese population (2, 7). The CUA of vaccines against COVID-19 in Hong Kong also did not consider the heterogeneity of the population (12).

**Figure 1:**
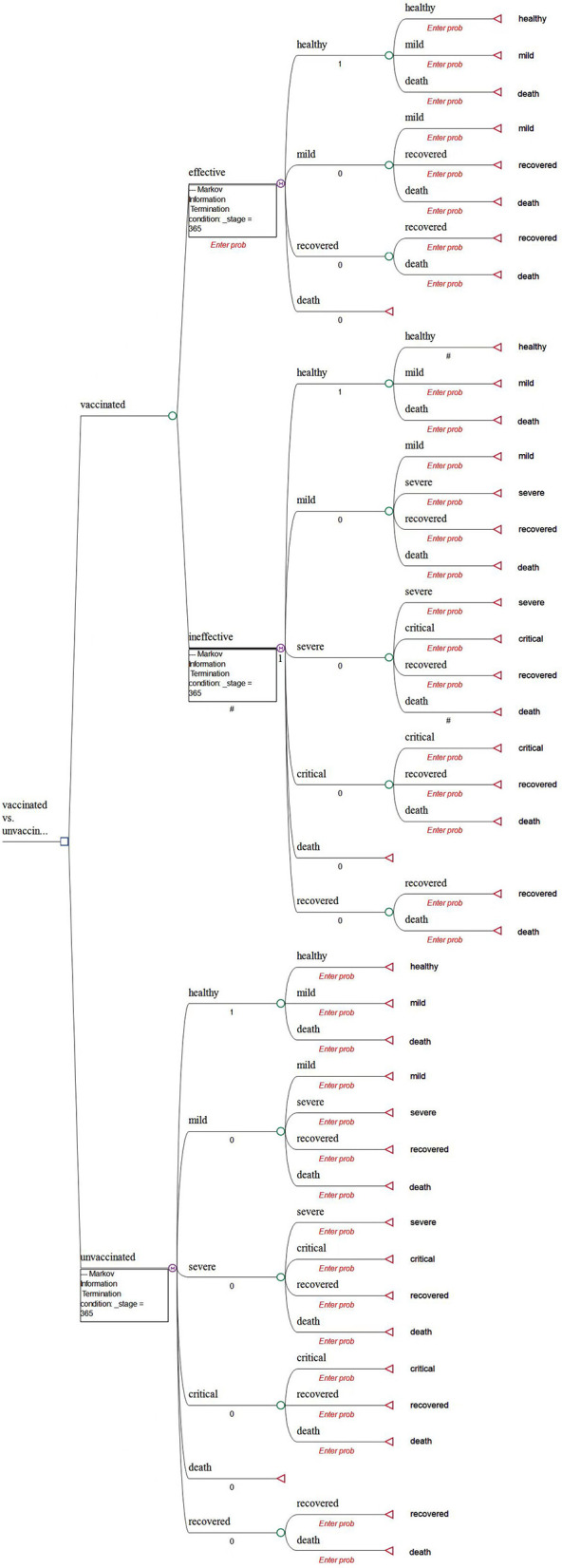
Decision tree model of “vaccinated” vs. “not vaccinated”.

In the model, all populations (both vaccinated and unvaccinated) are assumed to start from the “healthy” state (Figure 1). Populations in the vaccinated arm are assumed to have received two doses of inoculation and completed the immunogenic process before being classified into the “effectively vaccinated” arm or “ineffectively vaccinated” arm (Figure 1).

**Figure 2:**
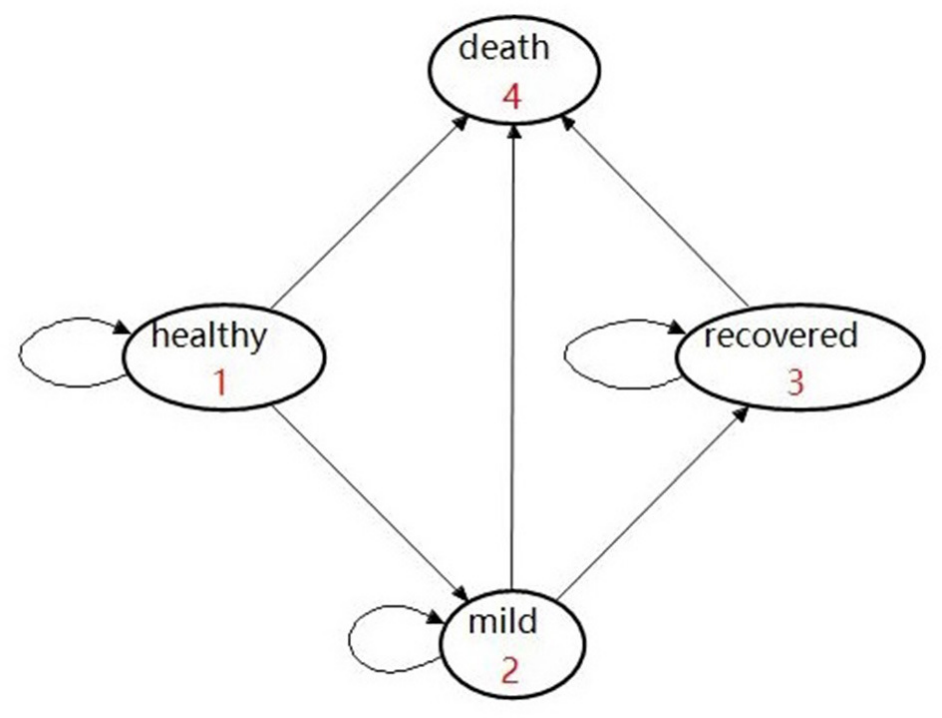
Markov health states showing an “effectively vaccinated” arm.

Given that vaccination can effectively reduce the rate of developing severe conditions, hospitalization, and death (7–9, 13), the possibility of prevention of hospitalization is used as the vaccine effectiveness in our study. Therefore, we assume that it would be impossible for the effectively vaccinated population to deteriorate into the “severe” or “critical” state due to COVID infection. In addition, a background mortality rate is assigned to the population. As a result, there are four health states, namely, healthy, mild, recovered, and deceased (Figure 2). In detail, individuals may be classified as being infected and then may develop mild symptoms in the first stage. Once they are infected and develop mild conditions, they could either move into the “recovered” state or they would maintain mild conditions in the next stage.

**Figure 3:**
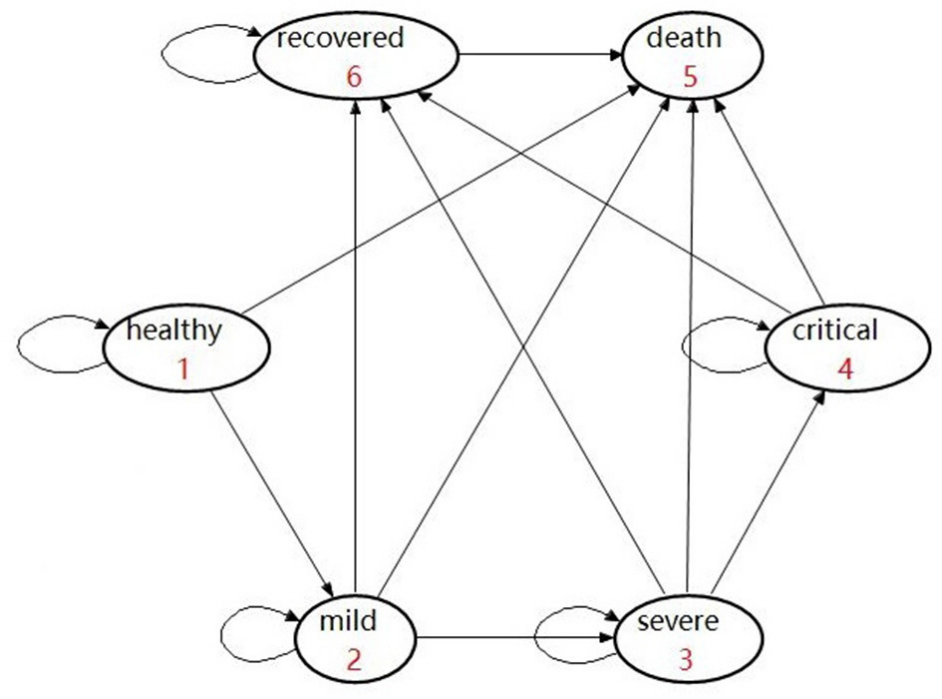
Markov health states showing the “ineffectively vaccinated” and “not vaccinated” arms.

In the model, individuals classified into the “ineffectively vaccinated” category are deemed unvaccinated. They were incorporated within another Markov model that consisted of six health states, namely, healthy, mild, severe, critical, recovered, and deceased (Figure 3). In detail, those who are unvaccinated or ineffectively vaccinated may develop mild, severe, and critical symptoms once they were infected, where the worst outcome would be death (due to COVID).

The cycle length of the Markov model is 1 day (14), and the time horizon of the CUA is 1 year (14, 15). This setting could capture the characteristics of symptom development of COVID-19 based on reported evidence. Other key assumptions are reported in Supplementary Table S1.

### 2.2. Model inputs

#### 2.2.1. Transition probabilities

As with previous health economic studies on vaccines against COVID-19 (4, 15), we do not explicitly distinguish efficacy and effectiveness in this study. As discussed previously, we use the possibility of hospitalization prevention as the parameter of vaccine effectiveness in the model. Its base case value (78.7%) is extracted from the WHO evidence assessment report of the Beijing unit of Sinopharm's China National Biotech Group (7).

As there is no existing data regarding the effectively vaccinated people's transition probability of switching from being with mild conditions to recovery, we estimate the probabilities from a randomized controlled trial (16) in which a combination of Traditional Chinese Medicine (Hua Shi Bai Du granule) plus standard Western medicine is used to treat COVID-19 patients with mild symptoms (16). We believe that this is a quite suitable source because (1) the trial is of high academic rigor and published in a credible peer-reviewed journal, (2) all the patients in the trial finally recovered, and (3) none of them deteriorated to critical or death (due to COVID). The latter two facts are quite consistent with our assumption that it would be impossible for effectively vaccinated groups to deteriorate from “healthy” to “severe” and “critical”. Details related to the estimation of transition probabilities in this category are reported in Supplementary material 3.1, Transition probabilities of the effectively vaccinated groups. Meanwhile, people in this category could die for reasons unrelated to COVID-19. Their background mortality rate is extracted from a published cost-effectiveness analysis of vaccination against COVID-19 in the US (15).

Transition probabilities of the ineffectively vaccinated and unvaccinated groups are extracted from two previous health economic studies. One study estimated the cost-effectiveness of nonpharmacological interventions, including hand hygiene and different surgical masks in the general population, using the COVID-19 Chinese patient characteristics data in 2019 (14); the other study estimated the clinical and economic impact of four different COVID-19 test strategies in Massachusetts, US, with calibrated data from China and the US (17).

#### 2.2.2. Health outcomes

We conduct a CUA in this study. Based on the China Guidelines for Pharmacoeconomic Evaluations (18), the quality of life with the health conditions in this study is measured by the quality-adjusted life years (QALYs) which are obtained from published COVID-19 health economics studies. According to the National Clinical Practice Guideline for COVID-19 (19) and the summary of case reports in China (2), individuals who tested positive for COVID-19 with symptoms, such as fever, cough, or pneumonia imaging, would move to the mild state. From the mild state, individuals who develop severe symptoms, such as dyspnea, blood oxygen saturation of <93%, or lung infiltrates of more than 50% within 24–48 h, would move to the severe state. Individuals who develop severe respiratory failure, septic shock, or multiple organ dysfunctions would move to the critical state or the deceased state. Given these, health utility values of mild, severe, and critical states are derived from data on the quality of life collected from patients who experienced similar disease symptoms (14, 15, 20). In detail, the utility values of the patients with influenza, Clostridium difficile infection, and those patients in intensive care units (ICUs) are used as the utility scores of the patients with mild, severe, and critical conditions in our study, respectively.

Similar to the study by Bagepally et al. (14), we assume that recovered individuals could return to a state of perfect health.. In addition, we also include the QALY loss due to premature death. We account for the lifetime QALY loss due to a premature death that occurred in the 1-year period. It is a reasonable conventional approach, as it has been applied in a few health economic studies. For instance, the lifetime QALY loss due to premature death is calculated using the same approach and added to the total costs at the end of the 1-year period in a health economic study regarding the cost-effectiveness of prevention policies against COVID-19 in the UK (20), from which the base case value of the QALY loss due to premature death in our study is derived. The details of the estimation can be seen in Supplementary material 4, Health state utility.

In addition, the number of people in each state at the end of the simulation is calculated. As a result, the effects of vaccination and no-vaccination strategies are reported as infection rate, death rate, and proportions of the population by health states.

#### 2.2.3. Costs

The model accounts for healthcare costs and productivity costs from a societal perspective. The price of vaccines in mainland China is uniformly 200 CNY (US$27.95, and the conversion in 2022 is 7.16 CNY to US$1) per dose across the country. Individuals are required to receive two doses of vaccines, which cost 400 CNY in total. Healthcare costs of infected states are derived from a burden-of-disease study in China (3) in which costs of diagnosis, inpatient care, medicines, treatments, and follow-up appointments are calculated. On average, a mild case costs 6,488.90 CNY in 17 days, a severe case costs 61,351.57 CNY in 31 days, and a critical case costs 176,744.05 CNY in 45 days. Given these, the daily cost of mild, severe, and critical states is 381.70 CNY, 1,979.08 CNY, and 3,927.65 CNY, respectively. Details are reported in Supplementary material 5.1, Healthcare cost.

Productivity costs of surviving cases are calculated based on the income loss due to illness. The cost of death is calculated as the income loss due to premature death based on a daily income loss estimated from personal disposable income (21). Details are reported in Supplementary material 5.2, Productivity losses.

#### 2.2.4. Discount rate

The Markov model is a dynamic model which distinguishes the effectiveness and costs occurring on day 1 from those that occur on day 2 (in this study, the cycle of our Markov model is 1 day). Therefore, discounting has to be applied, although the time horizon of this study is 1 year. This approach has been applied in previous studies based on the cost-effectiveness of vaccines against COVID-19 in the US (15, 22) and Denmark (4), in which both QALYs and costs are discounted over a time horizon within 1 year. A traditional half-cycle correction is used in the Markov model, and an annual discount rate of 5% is adopted in this CUA. We achieve this by using the “global discounting” function in the TreeAge Pro software which automatically converts the annual discounting rate into the corresponding daily discounting rate.

The input parameters discussed earlier are summarized in Table 1.

**Table 1:** Input parameters.

**Parameter**	**Base case value**	**Possible range in one-way deterministic sensitivity analysis**	**Probabilistic distribution**
**Transition probabilities, effective vaccinated, daily**			
Efficacy of vaccine	0.787 (7)	0.260 (7) to 1.1 (9, 23)	Beta
Remaining healthy	0.999 (14)	0.976 to 0.999 (14)	Beta
Healthy to deceased	0.00006 (15)	0.00006 (15) to 0.00009 (15)	Beta
Healthy to mild	0.00094^a^		Beta
Remaining mild, 0–10 days	0.940 (16)^b^	0.487 to 0.799 (16)^a^	Beta
Mild to recovered, 0–10 days	0.05994^c^		Beta
Remaining mild, after 10 days	0.487 (16)^b^	0.487 to 0.799 (16)^a^	Beta
Mild to recovered, after 10 days	0.51294^c^		Beta
Mild to deceased	0.00006 (15)	0.00006 (15) to 0.00009 (15)	Beta
**Transition probabilities, ineffective or not vaccinated, daily**			
Remaining healthy	0.87494^d^		Beta
Healthy to mild	0.125 (17)	0.015 (14) to 0.221 (17)	Beta
Healthy to deceased	0.00006 (15)	0.00006 (15) to 0.00009 (15)	Beta
Remaining mild	0.621 (17)		Beta
Mild to recovered	0.09484 (17)^e^		Beta
Mild to severe	0.284 (17)		Beta
Mild to deceased	0.00016 (15)	0.00010 (15) to 0.00033 (15)	Beta
Remaining severe	0.930^f^		Beta
Severe to recovered	0.063 (17)	0.004 (14) to 0.063 (17)	Beta
Severe to critical	0.006 (14)	0.003 (14) to 0.105 (17)	Beta
Severe to deceased	0.001 (17)		Beta
Remaining critical	0.980^g^		Beta
Critical to recovered	0.008 (17)^h^		Beta
Critical to deceased	0.012 (14)		Beta
**Heath state utility, QALY**			
Healthy, daily	0.00274 (14)	0.00251 (20) to 0.00274 (14)	Normal
Mild, daily	0.00222 (15)	0.00211 (14) to 0.00251 (20)	Normal
Severe, daily	0.00141 (15)	0.00090 (14) to 0.00141 (15)	Normal
Critical, daily	−0.00080 (14)	−0.00162 to 0.00003 (15)	Normal
Lifetime QALY loss due to premature death	8.80 (20)	4.40 (20) to 14.09 (24, 25)	Normal
**Cost, CNY**			
Vaccination, per dose	200 (26)	100 to 300 (26)	Gamma
Healthcare for mild, daily	381.70 (3)^i^	190.85 to 572.55 (3)^a^	Gamma
Healthcare for severe, daily	1,979.08 (3)^i^	989.54 to 2,968.624 (3)^a^	Gamma
Healthcare for critical, daily	3,927.65 (3)^i^	1,963.82 to 5,891.47 (3)^a^	Gamma
Productivity loss for a surviving case, daily	123.45 (21)^j^	61.73 to 276.16 (21)^a^	Gamma
Productivity loss for a death case, annual	186,240.21 (21, 24)^j^	0 to 416,618.88 (21, 24)^a^	Gamma

a Healthy to mild = 1-remaining healthy-healthy to death.

b Estimation processes are shown in Supplementary material 3.1.

c Mild to recovered = 1-remaining mild-mild to death.

d Remaining healthy = 1-healthy to mild-healthy to death.

e Mild to recovered = 1 remaining mild—mild to severe-mild to death.

f Remaining severe = 1-severe to recover-severe to critical -severe to deceased.

g Remaining critical = 1-critical to recovery-critical to deceased.

h Transition probability of critical to recovery was calculated by multiplying transition probabilities of critical to recuperation with recuperation to recovered, which were used in the citation.

i Estimation processes are shown in Supplementary material 5.1.

j Estimation processes are shown in Supplementary material 5.2.

### 2.3. Analysis

#### 2.3.1. Base case analysis

We report incremental cost-effectiveness ratios (ICERs), which are calculated by the difference in cost divided by the difference in QALYs (CNY/QALY), from a societal perspective. In accordance with the China Guidelines for Pharmacoeconomic Evaluations (18), we take a GDP per capita of 72,000 CNY (US$10,048.71) in China in 2020 (21) as the threshold of willingness-to-pay (WTP). We define vaccination strategy as “cost-effective” if its ICER is below WTP and as “cost-saving” if its ICER is minus.

#### 2.3.2. One-way sensitivity analysis

One-way deterministic sensitivity analysis is conducted to assess the influences of the uncertainty of each variable on the results (revealed by tornado diagrams). In detail, we change the efficacy of vaccines from 26% (the lower bound of the 95% CI of efficacy) (7) to the most optimistic situation with an efficacy of 100% in the sensitivity analysis (9, 23). Similarly, the transition probabilities and utility values are changed to the lowest and highest likely values reported in the previous studies.

Given that the costs of vaccination and healthcare for patients in mainland China are provided and paid by the government in accordance with uniform standards and there is very limited literature regarding the heterogeneities in costs in China, cost parameters are increased and decreased by 50% in the sensitivity analysis to test the reliability of the results. The income loss due to illness is changed to zero in the most conservative situation and further changed to the highest possible values estimated based on GDP per capita (21). In addition, we calculate the possible range of QALY loss due to premature death based on the life expectancy, and then, we use the range to determine the upper and lower bound of the possible values in the sensitivity analysis (25). Further details are reported in the Supplementary material.

#### 2.3.3. Probabilistic sensitivity analysis

Probabilistic sensitivity analysis (PSA) is applied to evaluate the effects of uncertainty of different variables on results. In the PSA, we assume that costs, utility values, and transition probabilities follow gamma, normal, and beta distribution, respectively (Table 1). In addition, 1,000 iterations are set in the Monte Carlo simulation to realize the random influences.

We arrange the structure of this study following the China Guidelines for Pharmacoeconomic Evaluations (18) and report the study according to the Consolidated Health Economic Evaluation Reporting Standards 2022 (CHEERS 2022) statement (27). The checklist of CHEERS has been added to Supplementary Table S12.

## 3. Results

### 3.1. Base case outcomes

**Figure 4:**
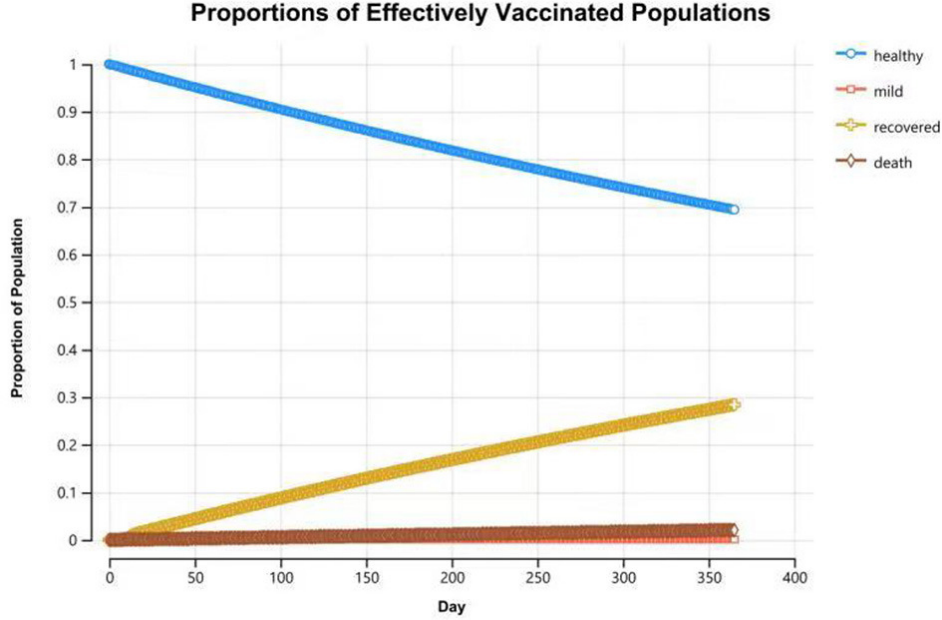
Proportions of populations by health states in the “effectively vaccinated” arm at the end of 1-year time horizon.

**Figure 5:**
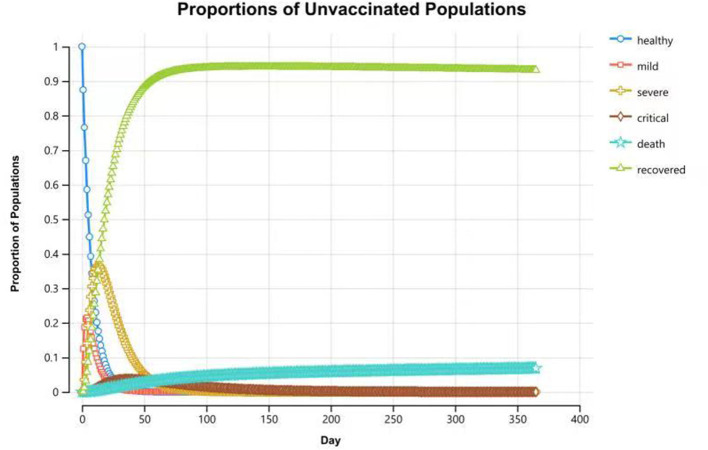
Proportions of populations by health states in the “ineffectively vaccinated” arm at the end of 1-year time horizon.

Compared with the unvaccinated scenario, vaccination would reduce the infection rate and mortality rate by a large margin. In the unvaccinated scenario, the probability of being infected is 100% in a year, and the death rate is 6.8%. Vaccination will decrease the infection rate to 45.3%, and the death rate will be reduced to 3.1%. Of the individuals who are effectively vaccinated, 69.4% of them will remain healthy and without infections at the end of the year, and the remainder (30.6%) will become infected and develop mild symptoms. Figures 4, 5 reveal the change in proportions of the population by health states (both “effectively vaccinated” and “ineffectively vaccinated” arms). Consequently, vaccination would yield a saving of 37,664.77 CNY (US$5,256.70) and a gain of 0.50 QALYs per person per year on average. The ICER was −74,895.69 CNY/QALY (US$-10,452.85). The results imply that vaccination is cost-saving compared with the no-vaccination scenario. The clinical outcomes and results in the base case are shown in Table 2.

**Table 2:** Clinical outcomes and cost-effectiveness results in base case.

	**Vaccinated**	**Nonvaccinated**
Infection rate, %	45.3	100
Death rate, %	3.1	6.8
Total cost, CNY	11,493.25	49,158.03
Total QALY	0.82	0.32
Incremental cost	−37,664.77	
Incremental QALY	0.50	
ICER, CNY/QALY	−74,895.69 (dominating)	

### 3.2. One-way sensitivity analysis

**Figure 6:**
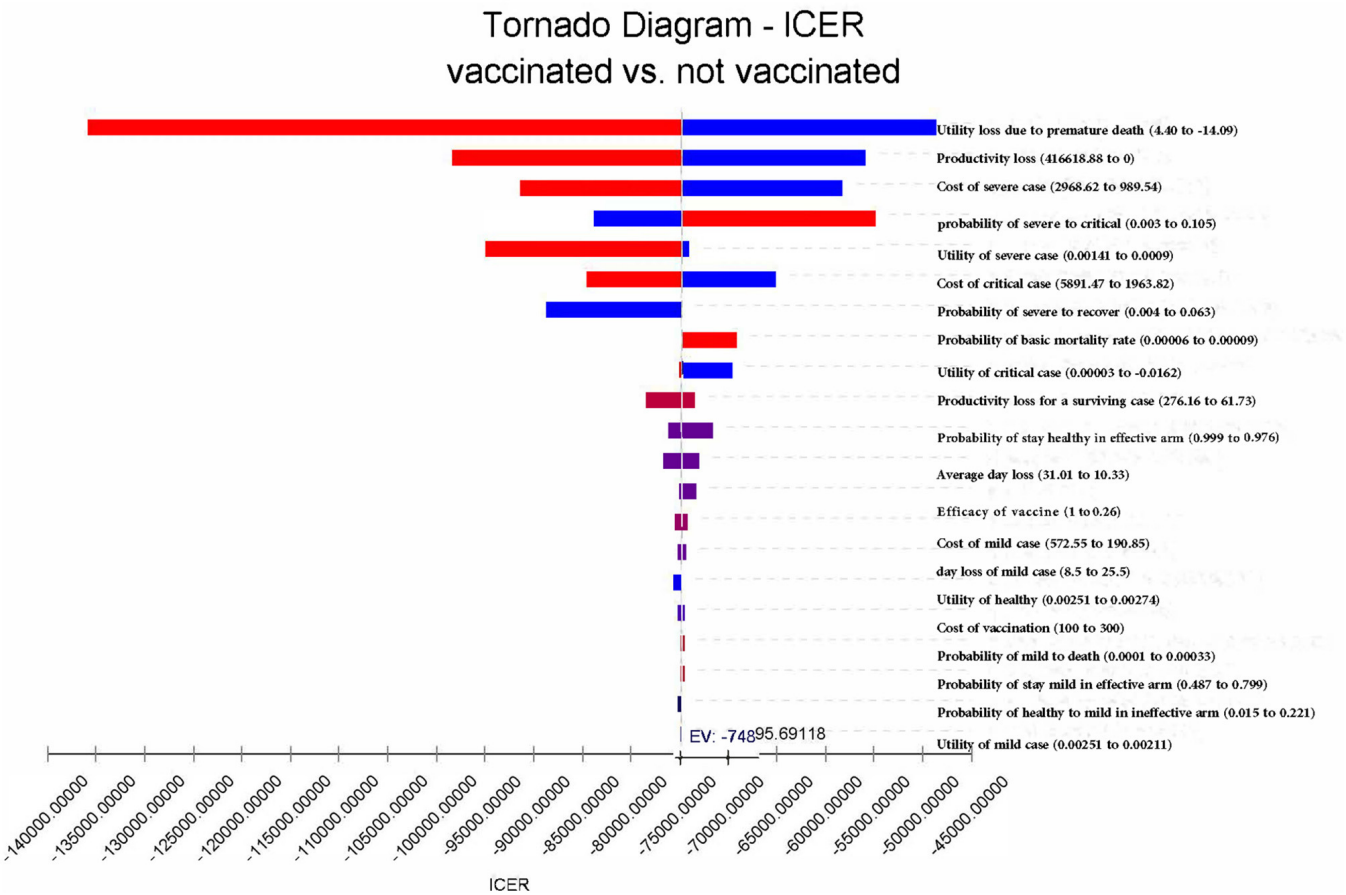
One-way sensitivity analyses for ICER of “vaccinated” vs. “not vaccinated”.

In summary, vaccination remains the dominating strategy, regardless of which parameters change to the extreme possible values. The result is relatively sensitive to utility loss and productivity loss due to premature death and the costs of critical cases (Figure 6). If productivity costs were not accounted for, vaccination would lead to a saving of 26,642.38 CNY (US$3,718.35). In this case, the ICER would change to −53,284.00 CNY/QALY (US$-7,436.60 per QALY) (Table 2). If the effectiveness of COVID-19 vaccines increased to 100%, the infection rate among the vaccinated population would reduce from 45.3% in the base case to 30.6%, and the mortality rate would decrease to 0. When adjusting the effectiveness of COVID-19 vaccines to 26.0%, vaccination would decrease the infection rate from 100 to 82.0%, and the mortality rate would be decreased from 6.8 to 5.0%. In this case, vaccination would lead to a saving of 12,175.40 CNY (US$1,699.26) and a gain of 0.17 QALYs per person per year on average.

### 3.3. Probabilistic sensitivity analysis

**Figure 7:**
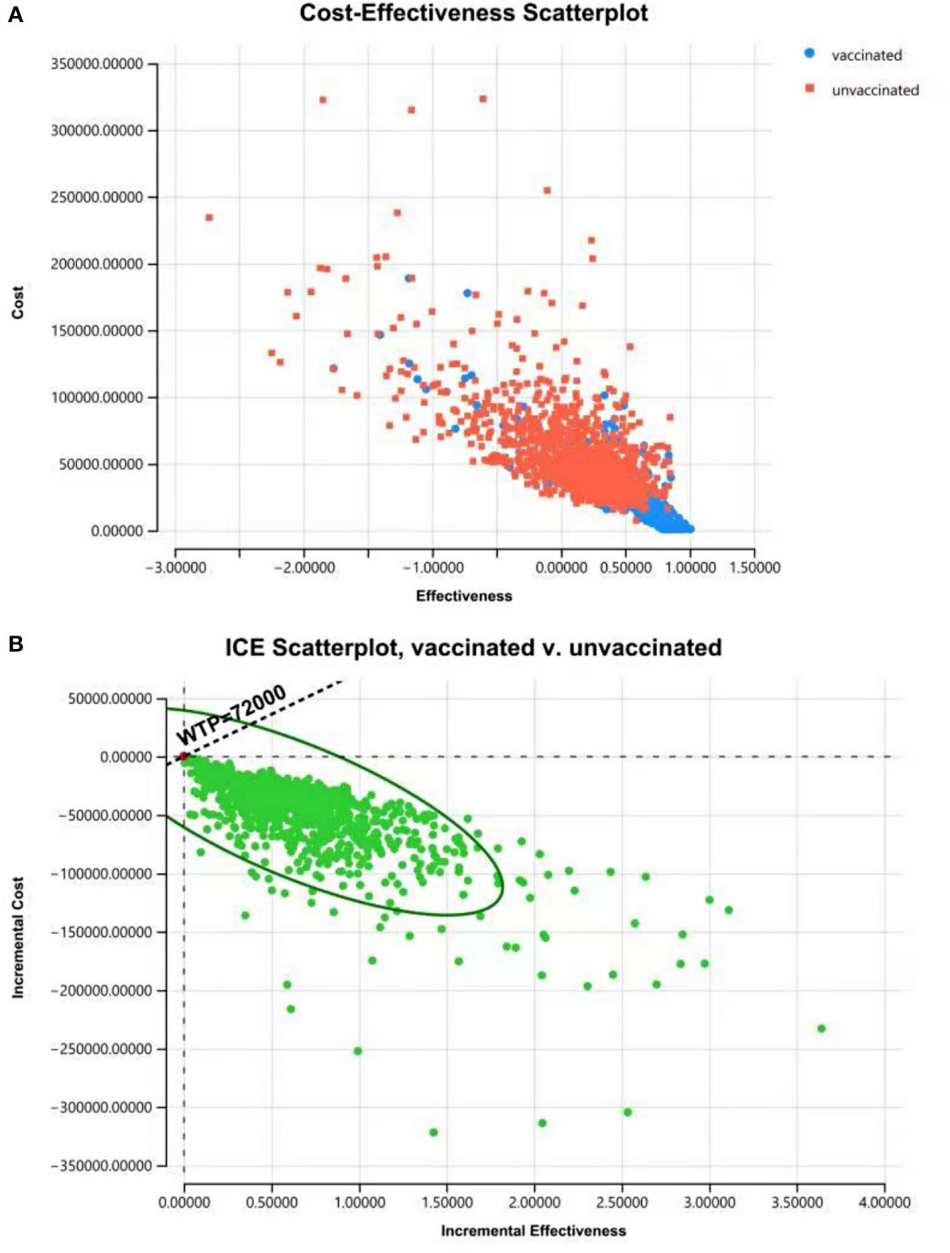
Cost-effectiveness plane for cost and effective **(A)** and ICER **(B)** of “vaccinated” vs. “unvaccinated” produced from the PSA, indicating that vaccination is more effective and less costly with a probability of 97.9% when the WTP is 72,000 CNY per QALY.

**Figure 8:**
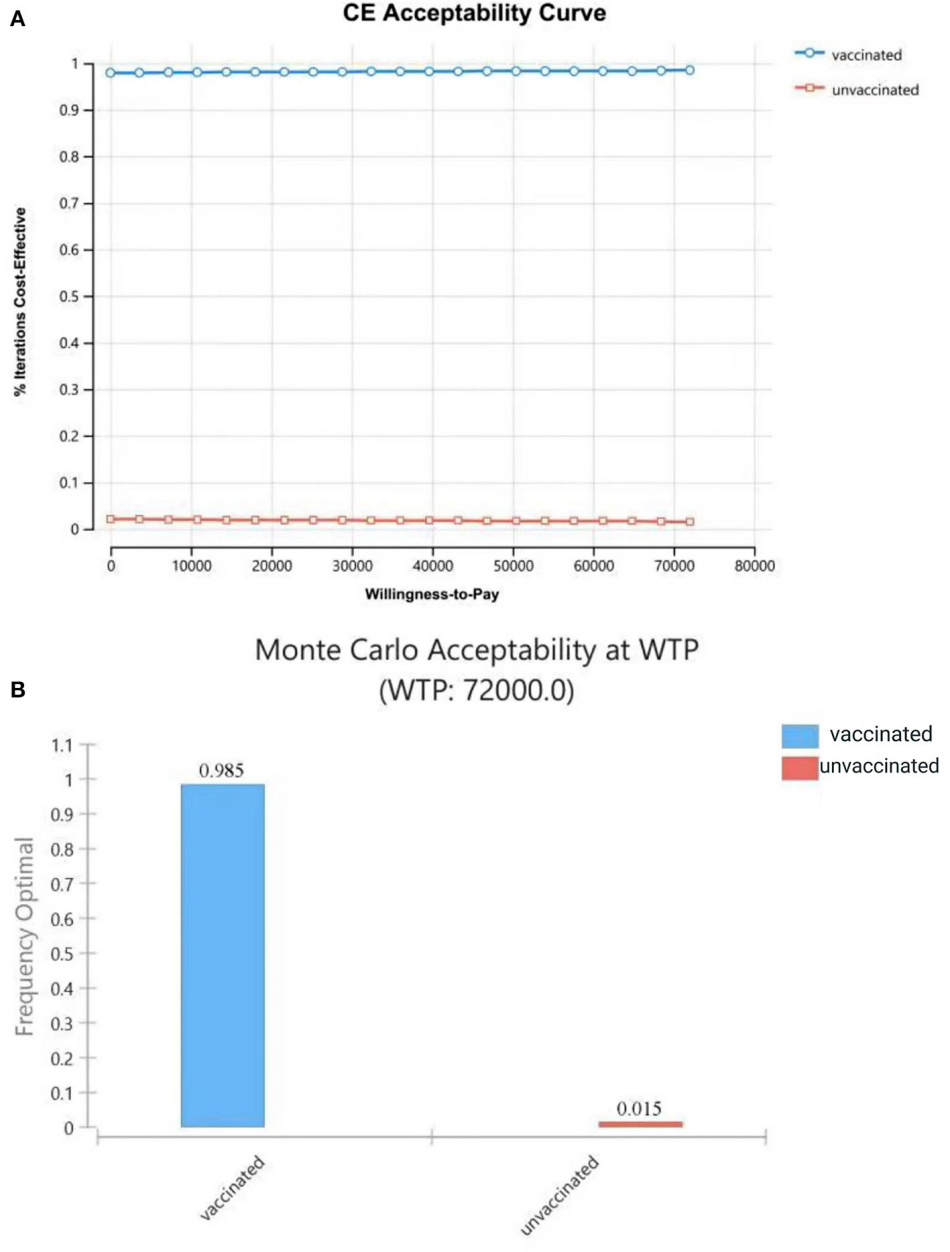
Cost-effectiveness acceptability curve **(A)** shows the probabilities of being cost-effective for “vaccinated” and “unvaccinated”, when the WTP ranges from 0 to 72,000CNY; **(B)** shows probabilities of being cost-effective when WTP is 72,000 CNY.

The results of PSA showed that most plots of the vaccination strategy are in the lower right of the plots of the unvaccinated (Figure 7A). Vaccination is more effective and less costly in 97.9% of the simulations in PSA when the WTP is 72,000 CNY per QALY (Figure 7B). The cost-effectiveness acceptability curve (Figure 8A) shows the probabilities of being cost-effective for “vaccinated” and “unvaccinated”, when the WTP ranges from 0 to 72,000 CNY. Figure 8B indicates that vaccination would maintain cost-effective with a probability is 98.5%, when the WTP is 72,000 CNY.

## 4. Discussion

In this study, we evaluate the cost-effectiveness of COVID-19 vaccines in China and find that compared with a laissez-faire scenario in which neither vaccination nor public restriction regulations are implemented, vaccination among the general population in China would reduce the infection rate and death rate of COVID by a large margin. The results remain stable after plausible changes are made to the model inputs, and the probability of vaccination to be the dominating strategy is 97.9%.

Although vaccination has been carried out intensively and widely in China, COVID-19 vaccines have not yet been introduced into the national vaccine programs. To make the best use of resources, we suggest that the economic efficacy of different COVID-19 vaccines should be assessed independently by both national health technology assessment agencies and third parties before vaccines are added to the programs (28). This study lays a sound foundation for the decision-making process in the government. It indicates that if Sinopharm/BBIBP COVID-19 inactivated vaccines are adopted, 54.7% of the population would be protected from COVID-19 infection, and the death rate will be reduced by 54.4%. As a result, vaccination would lead to a gain of 0.50 QALYs per person per year and a saving in healthcare expenditures of 26,642.38 CNY per person, as well as a reduction in a productivity loss of 11,022.39 CNY per person on average. In addition, it should be noted that there would be a higher health gain and a greater saving if CoronaVac (Sinovac Life Sciences), RBD-based protein subunit vaccines (ZF2001), and Ad5-nCoV vaccines (CanSino Biologics) are used, given that their efficacy (ranging from 87.5 to 100%) is much higher than the value of that used in the base case in this study (8, 9, 23, 29). The evidence above indicates that the Chinese government should insist on implementing a vaccination strategy in the future.

Moreover, we believe if people are inoculated with a third dose at 200 CNY, vaccination is very likely to remain the dominating strategy. The two reasons are as follows: (1) a third dose is very likely to generate additional health benefits since a phase-2 clinical trial has shown that it can create a remarkable increase in antibody levels, without causing any serious adverse event (30) and (2) in the sensitivity analysis, we have shown that the vaccination will remain as the dominating strategy when the cost of vaccines is increased by 50%. When the price is increased by 50%, the costs of 2 doses at the *ex post* price (300 CNY per jab, 600 CNY in total) are equal to the costs of 3 doses at the *ex ante* price (200 CNY per jab in the base case, 600 CNY in total). However, whether the three-dose vaccination strategy is more cost-effective than the two-dose vaccination strategy still needs to be explored in the future.

The findings in the study are thought to be very close to actual data in the real world. For instance, our model indicates that the infection rate of COVID-19 in 1 year is 100% for unvaccinated groups in a laissez-faire scenario. This is consistent with the obvious fact that COVID-19 is a highly transmissible disease, much more transmissible than SARS-CoV in 2003 and MERS-CoV in 2012 (2). The conclusion also coincides with an epidemic prediction using a dynamic mathematic model which reveals that the infection rate of SARSCoV-2 would be 100% if no disease-control strategy was introduced (31). Moreover, our model shows that of the individuals who are effectively vaccinated, 69.4% of them will remain healthy at the end of the year. This estimate is very close to the vaccine effectiveness of 65.9% in Chile in February 2021 (8). Finally, the death rate for unvaccinated people is 6.8% in our model, a value which is somewhat close to the COVID fatality rate of 2.3% in a case report in China in 2020 (24).

Moreover, the results of this study are somewhat consistent with the CUA of COVID-19 vaccination of some studies in other countries. Studies conducted in the UK (32) and Kenya (33) indicate that vaccination for the general population is cost-saving compared with the no-vaccination scenario. Studies conducted in the US indicate that vaccination for 60% general population (34) or for populations at risk, including healthcare providers and people aged over 65 years (15), is the dominating strategy, and vaccination involving other populations would be cost-effective (15, 34) compared with a no-vaccination scenario. Li et al. show that booster vaccinations for people aged over 65 years is cost-saving compared with the two-dose basic vaccination strategy, and the conclusion can be applied to a situation where Pfizer mRNA vaccines are used to against COVID-19 (22). Studies conducted in Hong Kong (12), Denmark (4), Colombia (35), and Pakistan (36) indicate that vaccination for the general population is cost-effective, given the WTP in certain contexts. The infection rate of the virus, cost of healthcare, and productivity loss may be the main influencing factors on the different results in different contexts. For instance, if the Omicron variant quickly spread in Hong Kong, rather than the Delta variant in the base case scenario, vaccination would become cost-saving (12). If the price of mRNA vaccines decreased from 500 DKK (US$68.96) to 300 DKK (US$41.38) in Denmark, vaccination would become cost-saving (4). Healthcare costs in Colombia (35) and Pakistan (36) are much lower than those costs in China and other contexts, which may lead to the ICER being cost-effective rather than cost-saving. When considering productivity loss from a societal perspective, vaccination would be more cost-effective than in a scenario only involving healthcare costs (4, 12). Those results are consistent with our study, which indicates that productivity loss is the most influencing factor in the one-way sensitivity analysis. In summary, CUAs of vaccination against COVID-19 in various contexts suggest that, compared with a no-vaccination scenario, vaccination would be a cost-effective strategy at least; when the costs of vaccines are low and vaccines are accessible to general population, there is a great possibility for a vaccination strategy to be dominating to a scenario without vaccination.

This study has some limitations. First, due to the lack of literature regarding the utility scores of patients with COVID-19, the health utilities in our model are extracted from the utilities of patients who experienced similar symptoms. Second, we simply assume that COVID survivors will recover and maintain a state of perfect health since there is very limited literature about the quality of life of COVID-19 survivors in the long term so far. Particularly, the assumption still needs to be verified in the future: it should be questioned how much proportion of survivors can fully recover, what percentage of them will have COVID sequelae, how severe these sequelae are, and how long these sequelae will last. Therefore, future studies should attempt to develop COVID-19-related utility scores from clinical trials with a long follow-up in which the long-term quality of life of survivors could be investigated. Third, we exclude the costs and QALY loss due to the isolation of the people who have a history of close contact with COVID-19 patients and suspected cases. If all of these were included, the benefits of vaccination would probably become more substantial. In addition, externalities from vaccination are not considered in this study. Vaccination is thought to have positive externalities in terms of protection for those unvaccinated populations and the benefits for the economy from easing public restrictions on economic activities (37, 38). If externalities were included, the benefits of vaccination would become larger. Finally, future studies should focus on the vaccination priority of sub-groups in China, since some studies have shown that it is more cost-effective to vaccinate the elderly population than younger adults in Denmark (4) and the US (14). However, given that the Chinese government has been extending vaccination programs in the general population aged over 3 years with adequate vaccine supply and that there is a lack of evidence in age-specific transition probabilities in the Chinese population, the current study is unable to conduct an age-stratified model.

## 5. Conclusion

Compared with a non-vaccination scenario, vaccination among the general population in mainland China will reduce the infection rate and death rate by a large margin, leading to a higher health gain and yielding a lower cost. Consequently, it is the dominating strategy from a societal perspective. The conclusions are considered robust in the sensitivity analyses.

## Data availability statement

The original contributions presented in the study are included in the article/Supplementary material, further inquiries can be directed to the corresponding author.

## Author contributions

HXZ and ND conceptualized the study. XH, ZL, HYZ, XJ, and JY collated the data. ND and HXZ analyzed the data and drafted the initial manuscript. ZL and WZ reviewed and revised the manuscript. All authors contributed to the interpretation of the results and article and approved the submitted version.
