# Supplementary material for: Cost-effectiveness analysis of vaccination against COVID-19 in China
Source: Front Public Health. 2023 Mar 7;11:1037556. doi: 10.3389/fpubh.2023.1037556 (PMC10027744; doi:10.3389/fpubh.2023.1037556)
Supplement: Supplementary file 1 [file Data_Sheet_1.PDF]

SUPPLEMENTARY MATERIAL

Cost-effectiveness analysis of vaccination against COVID-19 in China

Huixuan Zhou[1,2†], Ningxin Ding[3†*], Xueyan Han[4], Hanyue Zhang[5], Zeting Liu[6], Xiao Jia[2], Jingjing Yu[2], Wei Zhang[7]

[1] Department of Physical Fitness and Health, School of Sport Science, Beijing Sport University, Beijing, China
[2] Key Laboratory of Exercise and Physical Fitness, Ministry of Education, Beijing Sport University, Beijing, China
[3] School of Government, Wellington School of Business and Government, Victoria University of Wellington, Wellington, New Zealand
[4] School of Health Policy and Management, Chinese Academy of Medical Sciences & Peking Union Medical College, Beijing, China
[5] School of Physical Education, North East Normal University, Jilin, China
[6] Department of Mathematic Science, School of Sport Engineering, Beijing Sport University, Beijing, China
[7] Department of Chemical Drug Control, China National Institute for Food and Drug Control, Beijing, China
[†]These authors have contributed equally to this work and share first authorship
*** Correspondence:**
Ningxin Ding
dingning1@myvuw.ac.nz

Contents

1 Model overview and assumptions 2
2 Effective rates of vaccines 4
3 Transition Probabilities 4
3.1 Transition probabilities of the effectively vaccinated groups 4
3.2 Transition probabilities of the unvaccinated/ineffectively vaccinated group 7
4 Health State Utilities 9
5 Costs 10
5.1 Health care costs 10
5.2 Productivity losses 10
6 CHEERS 2022 checklist 11
Reference 15

Table of contents

Supplementary Table S1. Summary of Assumptions 2
Supplementary Table S2. Vaccination Effectiveness of the Products with Conditional Marketing Authorization in mainland China. 4
Supplementary Table S3. Number at risk in the treatment group during 16-day observation .. 5

Supplementary Table S4. Probability of maintain mild conditions per day estimated from regressions 6
Supplementary Table S5. Transition probabilities in the base case and one-way sensitivity analysis 8
Supplementary Table S6. Estimation of average age of infected individuals. 9
Supplementary Table S7. Health state utilities in base case and one-way sensitivity analysis . 9
Supplementary Table S8. Healthcare costs in base case and one-way sensitivity analysis 10
Supplementary Table S9. Average day loss of surviving cases in ineffectively vaccinated arm and unvaccinated arm 10
Supplementary Table S10. Productivity losses of surviving cases 11
Supplementary Table S11. Summary of productivity losses. 11
Supplementary Table S12. Consolidated Health Economic Evaluation Reporting Standards 2022 (CHEERS 2022) checklist (22). 11

Figure of contents

Supplementary Figure S1. Exponential regression of proportion at risk on the first 10 days ... 6
Supplementary Figure S2. Exponential regression of proportion at risk after 10 days 6
Supplementary Figure S3. Exponential function of proportion at risk from 0 to 16 days 7

1 Model overview and assumptions

There are two arms in our decision tree: vaccinated and unvaccinated arm (Figure 1). Populations in the vaccinated arm are assumed to have received two doses of inoculation and completed the immunogenic process before classified into the 'effectively vaccinated' arm or 'ineffectively vaccinated' branch (Figure 1). The effectively vaccinated group have four health states: healthy, mild, recovered, and death (Figure 2). By contrast, individuals classified into 'ineffectively vaccinated' category are incorporated within another Markov model that consisted of 6 health states: healthy, mild, severe, critical, recovered, and dead (Figure 3).

Model assumptions are summarized in Supplementary Table S1.

Supplementary Table S1. Summary of Assumptions

Inputs	Assumptions
Population	There is limited evidence about whether people who are highly likely to developing serious adverse events should receive vaccination or not (1). So far, studies have shown that severe and immediate allergic reactions are the only absolute contraindications of vaccination (1). In addition, data indicates that the supply of vaccines is adequate for a collective vaccination, and Chinese government does not have to determine the prioritization of vaccination by health risks like many western countries have been doing so (2). Given the safety and efficacy of the vaccines (3-6), Chinese populations

	aged > 3 have been encouraged to take a two-dose inoculation of inactivated vaccines since September 2020. Therefore, we assume that the individuals modeled in this study are general population in China aged above 3. In addition, we assume populations are homogeneous, so parameters do not vary with age, gender, or other physical characteristics.
Health states	Studies have shown that COVID-19 vaccines will still able to reduce severe cases, hospitalization, and death rate, even if virus variation occurs (3-5, 7). Given this, we assume that the effectively vaccinated group in the model have four health states: healthy, mild, recovered, and death (not COVID-related). In detail, all populations are assumed to start from the 'healthy' state. Individuals classified into this category may be infected and then, developing mild symptoms in each cycle. Once they were infected and developed mild conditions, they would have a chance of transferring to the 'recovered' state in the next cycle; or, alternatively, they would maintain with mild conditions in the next period.<br>In the model, individuals classified into 'ineffectively vaccinated' category are deemed as unvaccinated. They are incorporated within another Markov model that consisted of 6 health states: healthy, mild, severe, critical, recovered, and dead (Figure 3). By contrast, those who are either unvaccinated or ineffectively vaccinated may develop mild, severe, and critical symptoms once they were infected, and the worst outcome could be death (due to COVID).
Transition probabilities	Transition probabilities of the individuals classified as effectively vaccinated are extracted from a clinic trial where patients receive a combination treatment (8).
	Transition probabilities of the populations who are classified as either ineffective vaccinated or unvaccinated are derived from data collected both in China and US during the initial outbreak of COVID (9, 10) at a time when limited disease-control measures were implemented.
	Transition probabilities are assumed independent and remain constant during the one-year period.
Costs and QALY losses/gains	The direct healthcare costs include costs of vaccination, medical treatments, and productivity losses due to illness and death (both COVID-related and unrelated).<br>Non-direct healthcare costs, such as patients' family members' costs associated with vaccination, medical treatments, and quarantine, are not accounted for in this analysis.
	Given that severe adverse events are rarely observed and reported in clinical trials (4, 5, 11), we simply assume that

	vaccination will not cause severe adverse events, so no costs and QALY loss associated with adverse events are included in the model.
	Extra costs and QALY loss/gains from disease transmission (externalities) are not taken into account in this study, mainly because it is difficult to quantify the externalities associated with vaccination from other preventive activities, such as large-scale nucleic acid screening and public restrictions (12).

2 Effective rates of vaccines

The values of vaccination effectiveness are derived from the probabilities (efficacy) against severe cases and hospitalization. We believe it is appropriate because (1) studies have shown that vaccination is able to effectively reduce the risk of developing severe conditions, hospitalization, and death, and (2) efficacy and effectiveness are not usually distinguished in previous health economic studies on vaccines against COVID-19 usually explicitly. As reported in Supplementary Table S2, the efficacies against hospitalization range from 78.7% to 100%. In order to be conservative, the value of 78.7% is used in base case in this study, and the likely range is set to be 78.7% to 100% in the one-way deterministic sensitivity analysis.

Supplementary Table S2. Vaccination Effectiveness of the Products with Conditional Marketing Authorization in mainland China.

Vaccine		Efficacy against severe infection or hospitalization, %	Source
Inactivated vaccine	Beijing unit of Sinopharm's China National Biotech Group	78.7	(11)
	Wuhan unit of Sinopharm's China National Biotech Group	100.0	(5)
	Sinovac Biotech	87.5	(4)
Protein subunit vaccine	Anhui Zhifei Longcom Biophar maceutical Company	100.0	(13)
Adenovirus vaccine	CanSino, Beijing Institute of Biotechnology	90.1	(14)

3 Transition Probabilities

3.1 Transition probabilities of the effectively vaccinated groups

Studies have shown that vaccinated individuals are found to have a faster decline in viral load than the unvaccinated groups, and vaccination is probably a highly effective strategy for preventing severe cases and death (4, 5, 11, 15). However, data also

indicates that although it is less likely for vaccinated groups to develop to severe cases and death, fully vaccinated groups are still facing the risks of being infected by SARS-CoV-2, particularly when viral variants occur (15). Given the facts described above, we assume the healthy individuals classified as ‘effectively vaccinated’ in the Markov model may be infected in each cycle and then, developing mild symptoms, but it would be impossible for them to deteriorate to the ‘severe’, or ‘critical’ state or die due to COVID infection. Once they were infected and developed mild conditions, they would have a chance of transferring to the ‘recovered’ state in the next cycle; or, alternatively, they would maintain with mild conditions in the next period.

Since there is no existing data regarding the effectively vaccinated people’s transition probability of switching from being with mild conditions to recovery, we estimate the probabilities from a randomized controlled trial in which a combination of Traditional Chinese Medicine (Hua Shi Bai Du granule) plus standard western medicine are used to treat COVID-19 patients with mild symptoms (8). Huashi Baidu granule (Q-14) is a compound composed of 14 Chinese herbs, which has been recommended for the treatment of COVID-19 by the National Clinical Practice Guideline for COVID-19 in China (16). In a single-center, open-labeled RCT which included 149 patients, Liu et al. found that compared with the control group who received a standard care alone, treatment group treated by Q-14 plus standard care had a higher rate of symptoms disappearance, but no significant difference was found in the conversion time between those two groups. In this study, over 80% of patients were with mild symptoms at the beginning of the trial, and critical cases were excluded in the experiment. All patients in the trial recovered in 16 days, and nobody deteriorated from mild condition to critical condition. We believe this is a quite suitable source because the trial is of high academic rigor, and published on a credible peer-reviewed journal. Moreover, the observed facts discussed above are quite consistent with our assumption that it would be impossible for effectively vaccinated groups to deteriorate from ‘healthy’ to ‘severe’ and ‘critical’.

As shown in Supplementary Figure S1 and S2 below, having plotted the proportion of people at risk in this trial on a graph (Supplementary Table S3), two exponential regressions were applied: one was used to simulate the decay in people at risk in the first 10 days (Supplementary Figure S1), the other was applied to quantify the change in people at risk from day-11 to day-16 (Supplementary Figure S2). The exponential regression is considered appropriate and finally opted because daily transition probabilities are assumed to be constant in our Markov model. As it is shown in Supplementary figure 2, the fitness of the two regressions is $R^2=1$ and $R^2=0.9969$ respectively. We believe the excellent fitness verifies the regression format.

The two regression functions are then used to calculate the daily transition probability of maintaining mild conditions. As shown in Supplementary Table S4, the daily transition probability is 0.940 in the first 10 days (when x=1, $y=e^{0.062x}=0.94$), and 0.487 after ten days. These estimates are used in the base case.

Supplementary Table S3. Number at risk in the treatment group during 16-day observation

Time in days	Number at risk, n =71	Proportion at risk
0	71	1.00
10	38	0.54
12	10	0.14
14	4	0.06
16	0	0.00

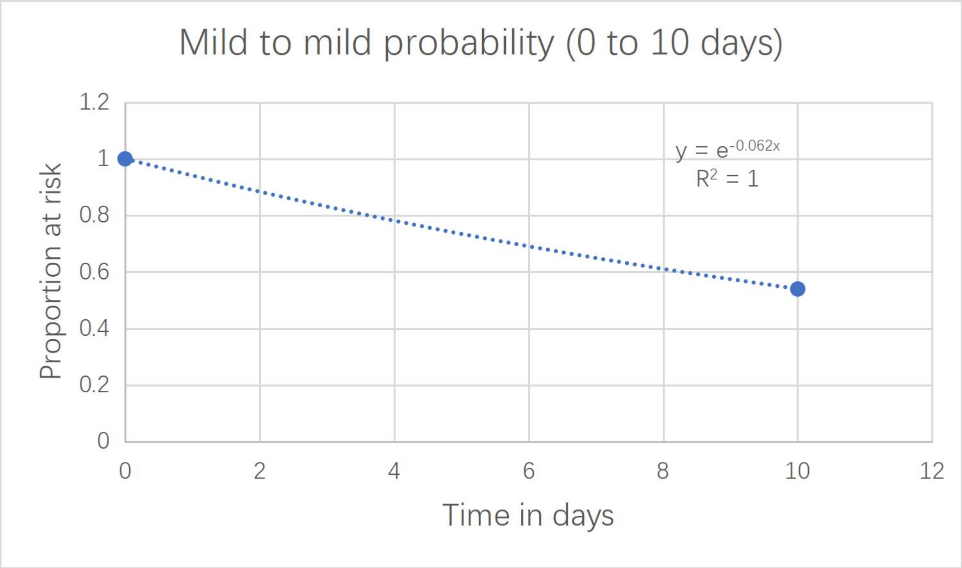

Supplementary Figure S1. Exponential regression of proportion at risk on the first 10 days

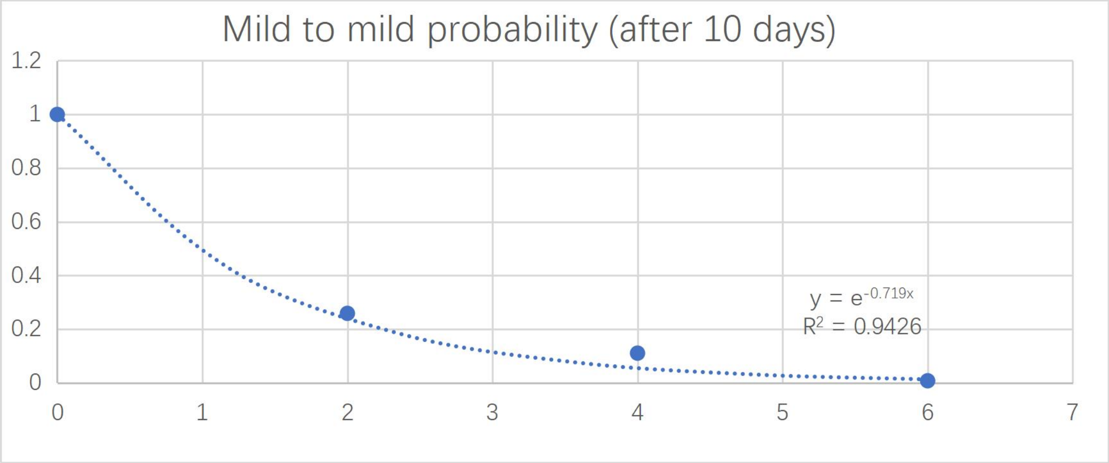

Supplementary Figure S2. Exponential regression of proportion at risk after 10 days

Supplementary Table S4. Probability of maintain mild conditions per day estimated from regressions

Scenarios	Probability of maintain mild conditions per day

Base case	0.94 (in the first 10 days)
	0.487 (after 10 days)
Sensitivity analysis	0.799

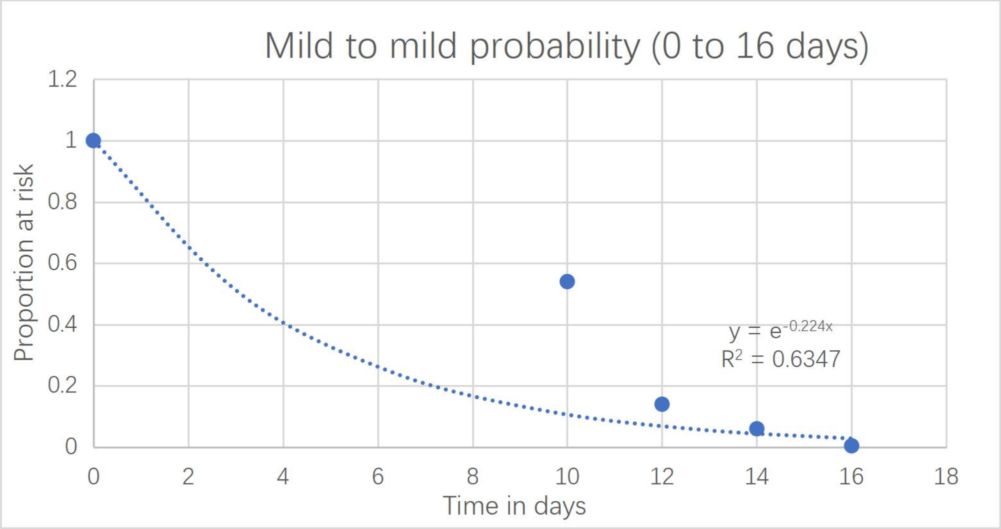

Supplementary Figure S3. Exponential function of proportion at risk from 0 to 16 days

The value of the daily transition probabilities of maintaining mild conditions in the sensitivity analysis is estimated using a similar approach. Having plotted the proportion of people at risk (Supplementary Table S3) on a graph (Supplementary Figure S3), an exponential regression was applied, and the regression function was then used to calculate the daily transition probability (daily transition probability of maintaining mild symptoms is 0.799). Given that the fitness of this regression is lower than the fitness of the two regressions above, we believe it is appropriate to use the estimate from this regression in the sensitivity analysis, and to use the estimates calculated from the two regressions above in the base case.

The transition probabilities of staying healthy are sourced from two literatures. One is from Bagepally et al.'s study in which the transitional probability of staying healthy is 0.999 (9); the other is from Neilan et al.'s study in which the transition probability of healthy to mild is 0.125 (17). The former value is used as the transition probability of staying healthy for the effectively vaccinated populations, and the latter is used as the transition probability of staying healthy for the ineffectively vaccinated and unvaccinated groups. We believe this design is appropriate because the latter one is smaller than its counterpart. The background death rate of this category is extracted from a cost-effectiveness analysis of vaccination against COVID-19 in US (18).

3.2 Transition probabilities of the unvaccinated/ineffectively vaccinated group

Transition probabilities of the patients who are either ineffectively vaccinated or unvaccinated are extracted from previous health economic studies in which transition probabilities are estimated from cases reports both in China and US (9, 10). As with the transition probabilities of the patients who are effective vaccinated, we assume

transitional probabilities of the patients who are either ineffectively vaccinated or unvaccinated remain as a constant within the one-year horizon. The values and sources of transition probabilities in the base case and one-way sensitivity analysis are summarized in Supplementary Table S5.

Supplementary Table S5. Transition probabilities in the base case and one-way sensitivity analysis

Transitional probability	Base case	Sensitivity analysis	
		Lower	Upper
Effective vaccinated arm, daily			
Efficacy of vaccine	0.787(11)	0.260 (3)	1(5, 13)
Stay healthy	0.999 (9)	0.976(9)	0.999(9)
Healthy to death	0.00006 (18)	0.00006(18)	0.00009(18)
Healthy to mild	0.00094[a]		
Stay mild, 0 to 10 days	0.940 (8)[b]	0.487(8)	0.799(8)
Mild to recovery, 0 to 10 days	0.05994[c]		
Stay mild, after 10 days	0.487 (8)[b]	0.487(8)	0.799(8)
Mild to recovery, after 10 days	0.51294[c]		
Mild to death	0.00006(18)	0.00006(18)	0.00009(18)
Ineffective vaccinated and unvaccinated arm, daily (10)			
Stay healthy	0.87494[d]		
Healthy to mild	0.125(17)	0.015(9)	0.221 (17)
Healthy to death	0.00006(18)	0.00006(18)	0.00009(18)
Stay mild	0.621 (17)		
Mild to recovery	0.09484 (17)[e]		
Mild to severe	0.284 (17)		
Mild to death	0.00016 (18)	0.00010(18)	0.00033(18)
Stay severe	0.930[f]		0.930 (9, 17)
Severe to recover	0.063 (17)	0.004 (9) to 0.063 (17)	0.063 (17)
Severe to critical	0.006 (9)	0.003 (9) to 0.105 (17)	0.006 (9)
Severe to die	0.001 (17)		0.001(17)
Stay critical	0.980[g]		
Critical to recovery	0.008 (17)[h]		
Critical to die	0.012 (9)		

[a] Healthy to mild =1-stay healthy-healthy to death. [b] Estimation processes are shown in the Supplementary Materials 3.1. [c] Mild to recovered=1-stay mild-mild to death. [d] Stay healthy=1-healthy to mild-healthy to death. [e] Mild to recovered=1- stay mild – mild to severe-mild to death. [f] Stay severe=1-severe to recover- severe to critical -severe to dead. [g] Stay critical=1-critical to recovery-critical to dead. [h] Transition probability of critical to recovery was calculated

by multiplying transition probabilities of critical to recuperation with recuperation to recovered, which were used in the citation.

4 Health State Utilities

Utility values used in this study are obtained from previous health economic studies. Utility values of mild, severe and critical states are derived from studies on patients' quality of life in which patients experienced symptoms similar to COVID-19 (9, 18, 19). And the value is changed 50% in the sensitivity analysis.

Unlike the base case value of QALY loss due to a premature death is soured from a previous study, the value used in the sensitivity analysis is estimated following the methods below. As shown in Supplementary Table S6, we start from calculating each sub-group's age (midpoint), one year after the simulation started (the time horizon of our Markov model is one year). And then, the average age of the whole groups is calculated as the weighted sum of the each sub-group's age (one year after the simulation started) (20). The result shows the average age of infected individuals after one year is around 53. Given that Chinese people's life expectancy is 78 (21), the years lose due to a premature death is 78-53=25. Having assumed the QALY loss of one year is 1 (QALY) and an annual discounting rate of 5%, we calculated the QALY loss of a premature death using to the equation:

$$QALY\ Loss = \sum_{i=1}^{i=25} 1/(1+0.05)^{\mathrm{i}} = 14.09.$$

As shown above, the QALY loss of a premature death is around 14.09. This value is used in the one-way sensitivity analysis, and the values of health utilities are summarized in Supplementary Table S7.

Supplementary Table S6. Estimation of average age of infected individuals.

Age range	Age (median) after 1 year	Proportion	Weighted
≥80	81	0.03	2.43
30-79	55.5	0.87	48.29
20-29	25.5	0.08	2.04
10-19	15.5	0.01	0.16
0-10	6	0.01	0.06
Average age			52.97

Supplementary Table S7. Health state utilities in base case and one-way sensitivity analysis

Health state utility, QALY	Base case	Sensitivity analysis	
		Lower	Upper
Healthy, daily	0.00274 (9)	0.00251 (19)	0.00274 (9)
Mild, daily	0.00222 (18)	0.00211 (9)	0.00251 (19)
Severe, daily	0.00140 (18)	0.00079 (9)	0.00140 (18)
Critical, daily	-0.00078 (9)	-0.00162(18)	0.00003 (18)

Lifetime QALY loss due to premature death	8.80 (19)	4.40 (19)	14.09 (20, 22)

5 Costs

5.1 Health care costs

Vaccine price of (200 CNY per dose) is used as the value of costs of vaccination in this study. And the value is then varied 50% lower and upper in one-way sensitivity analysis to treat uncertainties. Similarly, having obtained the value of healthcare costs for mild, severe, and critical cases from a cost-of-illness study in China (12), we calculated the daily costs for the each situation and then varied them by 50% in the sensitivity analysis, see Supplementary Table S8.

Finally, both costs and QALYs are discounted at the daily rate in this study (the 5% annual rate is converted to the corresponding daily rate by TreeAge Pro Healthcare).

Supplementary Table S8. Healthcare costs in base case and one-way sensitivity analysis

Health state	Total healthcare cost, CNY(12)	Course in days(12)	Base case healthcare cost, CNY	Sensitivity analysis	
				Lower, CNY	Upper, CNY
Mild	6,488.90	17	381.70 per day	190.85	572.55
Severe	61,351.57	31	1,979.08 per day	989.54	2,968.624
Critical	176,744.05	45	3,927.65 per day	1,963.82	5,891.468
Vaccinated			200 per dose	100	300

5.2 Productivity losses

Productivity losses due to illness are calculated by the methods described blow. Data indicates that it usually costs 17 days for an effectively vaccinated individual (mild case) to recover (12). Given the income per workday of 123.45 CNY (income per workday = annual income of (32,186/365) * (7/5) =123.45 CNY) (21), the productivity loss for a successfully vaccinated individual is calculated as 123.45 CNY *17=2,098.70 CNY.

As shown in Table S9 below, having calculated an unvaccinated individual's average day loss (20.67 days) as the weighted sum of day loss for each sub-group (mild, severe, and critical) (20), the productivity loss for an unvaccinated/ineffectively vaccinated individual is calculated as 20.67*123.45 CNY =2,551.78 CNY.

The value of upper boundary of income per work day in one-way sensitivity analysis is calculated by the same method, the only difference is the annual disposable income is replaced by per capital of GDP 72 000 CNY in 2020 (21). And the value of lower boundary is set to be 50% of the value used in the base case. Results are summarized in Supplementary Table S10.

Supplementary Table S9. Average day loss of surviving cases in ineffectively vaccinated arm and unvaccinated arm

Severity of case	Proportion (20)	Day loss (12)	Weighted day loss
Mild	0.80	17	13.60
Severe	0.14	31	4.28
Critical	0.06	45	2.79
Average day loss			20.67

Supplementary Table S10. Productivity losses of surviving cases

Base case	Day loss	Income per workday, CNY	Productivity loss per case, CNY
Mild case in effectively vaccinated arm	17	123.45	2 098.70
Surviving case in ineffective vaccinated and unvaccinated arm	20.67	123.45	2 551.78
Sensitivity analysis			
Mild case in effectively vaccinated arm	17	276.16	4 694.79
Surviving case in ineffective vaccinated and unvaccinated arm	20.67	276.16	5 708.32

Given that the expected age at death is 53 (see Supplementary Table S6) and the official age at retirement (60 in mainland China), an individual is expected to yield a 7-year income loss if the person is dead during the one-year period simulated in the Markov model. Therefore, the productivity loss due to a pre-mature death is calculated as the summation of discounted annual disposable income during the 7-year period (annual discounting rate is 5%):

$$Productivity\ loss = \sum_{i=1}^{i=7} Annual\ income/\ (1+0.05)^{\mathrm{i}}$$

The results indicate that when annual income is CNY 32,186, the value of productivity loss is CNY 186,230.21. In the sensitivity analysis, we switch to estimate the productivity loss based on GDP per capital. Calculation shows that when the annual income is replaced by GDP per capital of CNY 72,000, the value of productivity loss is CHY 30,653.33 (Supplementary Table S11).

Supplementary Table S11. Summary of productivity losses.

Productivity loss	Base case, CNY	Sensitivity analysis	
		Lower	Upper
Surviving case, daily	123.45	61.73	276.16
Death case, annual	186 240.21	0	416 618.88

6 CHEERS 2022 checklist

Supplementary Table S12. Consolidated Health Economic Evaluation Reporting

Standards 2022 (CHEERS 2022) checklist (23).

	Item	Guidance for Reporting	Reported in section
TITLE			
Title	1	Identify the study as an economic evaluation and specify the interventions being compared.	Intervention and economic evaluation have been specified in title of the main text.
ABSTRACT			
Abstract	2	Provide a structured summary that highlights context, key methods, results and alternative analyses.	Abstract includes context, methods, results from basic and alternative analyses.
INTRODUCTION			
Background and objectives	3	Give the context for the study, the study question and its practical relevance for decision making in policy or practice.	Context is reported in paragraph 1-3. The study question and practice are reported in paragraph 4 in 1 Introduction.
METHODS			
Health economic analysis plan	4	Indicate whether a health economic analysis plan was developed and where available.	2.1 Model overview.
Study population	5	Describe characteristics of the study population (such as age range, demographics, socioeconomic, or clinical characteristics).	Second paragraph in 2.1 Model overview.
Setting and location	6	Provide relevant contextual information that may influence findings.	Paragraph 4 in 1 Introduction.
Comparators	7	Describe the interventions or strategies being compared and why chosen.	First paragraph in 2.1 Model overview.
Perspective	8	State the perspective(s) adopted by the study and why chosen.	Paragraph 4 in 1. Introduction.
Time horizon	9	State the time horizon for the study and why appropriate.	Last paragraph in 2.1 Model overview.

Discount rate	10	Report the discount rate(s) and reason chosen.	2.2.4 Discount Rate.
Selection of outcomes	11	Describe what outcomes were used as the measure(s) of benefit(s) and harm(s).	2.2.2 Health outcomes
Measurement of outcomes	12	Describe how outcomes used to capture benefit(s) and harm(s) were measured.	2.2.2 Health outcomes.
Valuation of outcomes	13	Describe the population and methods used to measure and value outcomes.	2.3.1 Base case analysis.
Measurement and valuation of resources and costs	14	Describe how costs were valued.	2.2.3 Costs.
Currency, price date, and conversion	15	Report the dates of the estimated resource quantities and unit costs, plus the currency and year of conversion.	First paragraph in 2.2.3 Costs.
Rationale and description of model	16	If modelling is used, describe in detail and why used. Report if the model is publicly available and where it can be accessed.	Paragraph 3-5 in 2.1 Model overview.
Analytics and assumptions	17	Describe any methods for analyzing or statistically transforming data, any extrapolation methods, and approaches for validating any model used.	2.2.1 Transition probabilities, 2.2.2 Health outcomes and 2.2.3 Costs.
Characterizing heterogeneity	18	Describe any methods used for estimating how the results of the study vary for sub-groups.	The explanation can be seen in the second paragraph in 2.1 Model Overview.
Characterizing distributional effects	19	Describe how impacts are distributed across different individuals or adjustments made to reflect priority populations.	The explanation can be seen in the second paragraph in 2.1 Model Overview and the third paragraph in 1 Introduction.
Characterizing uncertainty	20	Describe methods to characterize any sources of uncertainty in the analysis.	2.3.2 One-way sensitivity analysis; 2.3.3 Probabilistic sensitivity analysis

Approach to engagement with patients and others affected by the study	21	Describe any approaches to engage patients or service recipients, the general public, communities, or stakeholders (e.g., clinicians or payers) in the design of the study.	Not available.
RESULTS			
Study parameters	22	Report all analytic inputs (e.g., values, ranges, references) including uncertainty or distributional assumptions.	Table 1.
Summary of main results	23	Report the mean values for the main categories of costs and outcomes of interest and summarize them in the most appropriate overall measure.	Table 2 and 3.1 Base Case Outcomes
Effect of uncertainty	24	Describe how uncertainty about analytic judgments, inputs, or projections affect findings. Report the effect of choice of discount rate and time horizon, if applicable.	3.2 One-way Sensitivity Analysis and 3.3 Probabilistic Sensitivity Analysis
Effect of engagement with patients and others affected by the study	25	Report on any difference patient/service recipient, general public, community, or stakeholder involvement made to the approach or findings of the study	Fifth paragraph in 4 Discussion.
DISCUSSION			
Study findings, limitations, generalizability, and current knowledge	26	Report key findings, limitations, ethical or equity considerations not captured, and how these could impact patients, policy, or practice.	4 Discussion.
OTHER RELEVANT INFORMATION			
Source of funding	27	Describe how the study was funded and any role of the funder in the identification, design, conduct, and reporting of the analysis	The source of funding for study authors are listed at the end of the manuscript. The funders had no role in identification, design, conduct, and reporting of the analysis.

Conflicts of interest	28	Report authors conflicts of interest according to journal or International Committee of Medical Journal Editors requirements.	A declaration of conflicts of interest is provided at the end of the text.

Reference

1. Yang S, Li Y, Dai L, Wang J, He P, Li C, et al. Safety and immunogenicity of a recombinant tandem-repeat dimeric RBD-based protein subunit vaccine (ZF2001) against COVID-19 in adults: two randomised, double-blind, placebo-controlled, phase 1 and 2 trials. The Lancet Infectious diseases. 2021;21(8):1107-19.
2. SohuNews. China approves 7 vaccines against COVID-19 and supplies 5 billion doses per year 2021 [Available from: https://www.sohu.com/a/478393338_121015503.
3. WHO. Evidence Assessment: Sinopharm/BBIBP COVID-19 vaccine. 2021. Contract No.: 4 May 2021.
4. Jara A, Undurraga EA, González C, Paredes F, Fontecilla T, Jara G, et al. Effectiveness of an Inactivated SARS-CoV-2 Vaccine in Chile. N Engl J Med. 2021;385(10):875-84.
5. Al Kaabi N, Zhang Y, Xia S, Yang Y, Al Qahtani MM, Abdulrazzaq N, et al. Effect of 2 inactivated SARS-CoV-2 vaccines on symptomatic COVID-19 infection in adults: a randomized clinical trial. Jama. 2021;326(1):35-45.
6. Han B, Song Y, Li C, Yang W, Ma Q, Jiang Z, et al. Safety, tolerability, and immunogenicity of an inactivated SARS-CoV-2 vaccine (CoronaVac) in healthy children and adolescents: a double-blind, randomised, controlled, phase 1/2 clinical trial. The Lancet Infectious diseases. 2021.
7. Singanayagam A, Hakki S, Dunning J, Madon KJ, Crone MA, Koycheva A, et al. Community transmission and viral load kinetics of the SARS-CoV-2 delta (B.1.617.2) variant in vaccinated and unvaccinated individuals in the UK: a prospective, longitudinal, cohort study. The Lancet Infectious Diseases. 2021;21(12):e363.
8. Liu J, Yang W, Liu Y, Lu C, Ruan L, Zhao C, et al. Combination of Hua Shi Bai Du granule (Q-14) and standard care in the treatment of patients with coronavirus disease 2019 (COVID-19): A single-center, open-label, randomized controlled trial. Phytomedicine : international journal of phytotherapy and phytopharmacology. 2021;91:153671.
9. Bagepally BS, Haridoss M, Natarajan M, Jeyashree K, Ponnaiah M. Cost-effectiveness of surgical mask, N-95 respirator, hand-hygiene and surgical mask with hand hygiene in the prevention of COVID-19: Cost effectiveness analysis from Indian context. Clinical epidemiology and global health. 2021;10:100702.
10. Neilan AM, Losina E, Bangs AC, Flanagan C, Panella C, Eskibozkurt GE, et al. Clinical Impact, Costs, and Cost-Effectiveness of Expanded SARS-CoV-2 Testing in Massachusetts. Clinical infectious diseases : an official publication of the Infectious Diseases Society of America. 2020.
11. WHO. Evidence Assessment: Sinopharm/BBIBP COVID-19 vaccine. 2021 May 4, 2021.
12. Jin H, Wang H, Li X, Zheng W, Ye S, Zhang S, et al. Economic burden of COVID-19, China, January-March, 2020: a cost-of-illness study. Bulletin of the World Health Organization. 2021;99(2):112-24.

13. SinaNews. China's protein subunit vaccine showed efficacy of 81.76% for prevention COVID-19, and 77.54% for Delta variant 2021 [Available from: https://news.sina.com.cn/c/2021-08-28/doc-iktzscyx0875449.shtml.
14. ObserverWeb. CanSino Bio: The conditional marketing application for adenovirus COVID-19 vaccine was accepted by the National Food and Drug Administration 2021 [Available from: https://www.360kuai.com/pc/9d5c52c75902e3cc5?cota=3&kuai_so=1&tj_url=so_vip&sign=360_57c3bbd1&refer_scene=so_1.
15. Singanayagam A, Hakki S, Dunning J, Madon KJ, Crone MA, Koycheva A, et al. Community transmission and viral load kinetics of the SARS-CoV-2 delta (B.1.617.2) variant in vaccinated and unvaccinated individuals in the UK: a prospective, longitudinal, cohort study. The Lancet Infectious Diseases.
16. NHC-China. National Clinical Practice Guideline for COVID-19 in China (Trial Eighth Edition). 2020 August 18.
17. Neilan AM, Losina E, Bangs AC, Flanagan C, Panella C, Eskibozkurt GE, et al. Clinical Impact, Costs, and Cost-Effectiveness of Expanded SARS-CoV-2 Testing in Massachusetts. Clinical infectious diseases : an official publication of the Infectious Diseases Society of America. 2020;73(9):e2908-e17.
18. Kohli M, Maschio M, Becker D, Weinstein MC. The potential public health and economic value of a hypothetical COVID-19 vaccine in the United States: Use of cost-effectiveness modeling to inform vaccination prioritization. Vaccine. 2021;39(7):1157-64.
19. Zala D, Mosweu I, Critchlow S, Romeo R, McCrone P. Costing the COVID-19 pandemic: an exploratory economic evaluation of hypothetical suppression policy in the United Kingdom. Value in health : the journal of the International Society for Pharmacoeconomics and Outcomes Research. 2020;23(11):1432-7.
20. Wu Z, McGoogan JM. Characteristics of and important lessons from the Coronavirus Disease 2019 (COVID-19) outbreak in China: summary of a report of 72 314 cases from the Chinese Center for Disease Control and Prevention. Jama. 2020;323(13):1239-42.
21. China NBoSo. China Statistical Yearbook: China Statistics Press; 2020.
22. NHC-China. Statistical Bulletin on Health Development 2019 2020 [Available from: http://www.nhc.gov.cn/guihuaxxs/s10748/202006/ebfe31f24cc145b198dd730603ec4442.shtml.
23. Husereau D, Drummond M, Augustovski F, de Bekker-Grob E, Briggs AH, Carswell C, et al. Consolidated Health Economic Evaluation Reporting Standards 2022 (CHEERS 2022) statement: updated reporting guidance for health economic evaluations. Bmj. 2022;376:e067975.
